
JOURNAL INFORMATION
==============================
NLM Title Abbreviation: Behav Neurol
Journal ID: Behav Neurol
NLM Journal ID: 8914585
Journal ID: BN

Behavioural Neurology

ISSN: 0953-4180
EISSN: 1875-8584
Publisher: Wiley

ARTICLE INFORMATION
==============================
PMCID: PMC5434418
PMID: 21422552
DOI: 10.3233/BEN-2010-0292
Article ID: 141082
Article version: 1
Subjects: Other

Dual Task Performance in Children and Adolescents with ADHD

Inasaridze Ketevan *
Bzhalava Vera
*Ketevan Inasaridze: kate_inasaridze@hotmail.com
Print publication date: 2010
Electronic publication date: 2011 Mar 7
Volume: 23
Issue: 4
First page: 193
Last page: 194
Received 2011 Jan 31; Accepted 2011 Jan 31
Copyright: Copyright © 2010 Hindawi Publishing Corporation and the authors.
Copyright year: 2010
License: This is an open access article distributed under the Creative Commons Attribution License, which permits unrestricted use, distribution, and reproduction in any medium, provided the original work is properly cited.
License URL: https://creativecommons.org/licenses/by/3.0/

==============================
Behavioural Neurology 23 (2010) 193–194
DOI 10.3233/BEN-2010-0292
IOS Press

### Dual task performance in children and adolescents with ADHD

Ketevan Inasaridze* and Vera Bzhalava

The deficit of executive functioning was found to be associated with attention deficit hyperactivity disorder (ADHD) in general and its subtypes [2,8]. One of the important functions of central executive is the ability simultaneously coordinate two tasks.

### 1. Objectives

The study aimed at defining the dual-task performance characteristics in healthy children/adolescents on the computerised and the paper and pencil dual-task methods; investigating the effect of task difficulty on dual-task performance; testing if the paper and pencil version of the dual-task method is giving the same results in ADHD and healthy controls; investigating whether the dual-task functioning in ADHD is defined by the general motor functioning and comorbidity factors.

### 2. Method

The study investigated dual-task functioning in 6–16 years old 91 typically developing controls and 91 children with ADHD. The 91 children with ADHD have been diagnosed in accordance with the DSM-IV criteria for ADHD, its subtypes (ADHD-C, ADHD-H and ADHD-I) and of comorbid disorders supplemented by the information from the Parent/Teacher Disruptive Behavior Disorder (DBD) Rating Scale [7]. The normal controls (NC) had a mean of DBD 23.50 (SD = 13.11) and children/adolescents with ADHD had a mean of DBD 64.25 (SD = 20.17). The 91 NC children were matched as closely as possible to 91 children/adolescents with ADHD group for age and years of education. The healthy controls had a mean age of 9.65 (SD = 2.66) and children/adolescents with ADHD had a mean age of 9.73 (SD = 2.68). The NC had a mean years of education of 4.46 (SD = 2.65) and ADHD group had a mean years of education of 4.46 (SD = 2.66). There was no statistically significant difference between the total raw scores of WISC-III for NC (mean = 269.77, SD = 73.17) and ADHD groups (mean = 252.01, SD = 81.34), t(175) = 1.525, $p = 0.129$. The significantly more boys with ADHD (64%) than NC boys (46%) participated in the study ($\chi^2 = 5.682$, df = 1, $p < 0.017$).

The experimental material included computerized [3] and paper and pencil ("Tbilisi paper and pencil motor task") versions [4] of the dual-task. The List Memory Task and computerized tracking task or paper and pencil motor tracking tasks were performed singly and then simultaneously. To investigate the task difficulty effect on ADHD in dual-task paradigm three difficulty levels were defined for the verbal and the computerized motor tasks [6]. To account for the overall changes in performance across both tasks the combined $\mu$ scores were calculated for each participant [7].

### 3. Results

For the computerised motor tracking there was the significant effect of type of task, F(1,179) = 27.187, $MSE = 21.776$, $p < 0.0001$, $\eta^2 = 0.132$; of group F(1,179) = 5.642, $MSE = 158.564$, $p < 0.018$, $\eta^2 = 0.031$ and an interaction F(1,179) = 4.298, $MSE = 21.776$, $p < 0.04$, $\eta^2 = 0.023$. For the List Memory Task the ANOVA showed a significant effect of type of task F(1,179) = 22.88, $MSE = 0.009$, $p < 0.0001$, but

*Corresponding author. E-mail: kate_inasaridze@hotmail.com.

ISSN 0953-4180/10/$27.50 © 2010 – IOS Press and the authors. All rights reserved no effect of group ($F < 1$) and no interaction ($F < 1$). The $\mu$ score for NC group (mean = 101.51, SD = 10.34) was higher in comparison to the group with ADHD (mean = 98.43, SD = 12.01), but was significant at one tail (t(179) = 1.847, $p < 0.066$). For the paper and pencil motor tracking task and the List Memory Task the ANOVA didn't showed a significant effect of type of task, of group or interaction and no significant difference was found in $\mu$ scores for the study groups. The subtypes of ADHD with or without comorbidity disorders didn't differ in dual task performance.

The moderate magnitude significant test-retest reliability was found for the computerised dual-task $\mu$ scores ($r = 0.36$, $p < 0.0001$) and for the paper and pencil dual-task $\mu$ scores ($r = 0.37$, $p < 0.0001$). For the NC group moderate magnitude significant test-retest reliability was revealed for the computerised $\mu$ scores ($r = 0.50$, $p < 0.0001$) and for the paper and pencil $\mu$ scores ($r = 0.39$, $p < 0.0001$). For the ADHD group the slightly low magnitude but significant test-retest reliability was found for the computerised $\mu$ scores ($r = 0.24$, $p < 0.025$) and for the paper and pencil $\mu$ scores ($r = 0.33$, $p < 0.001$). The correlation between the $\mu$ scores of the computerised and paper and pencil versions of the dual-task was defined ($r = 0.26$, $p < 0.0001$) to find out whether these two versions of the dual task are comparable counterparts of each other.

The general motor functioning assessed by the Movement Assessment Battery for Children–Second Edition (MABC-2) revealed significantly better balance (t(170.499) = 2.713, $p < 0.007$) in NC (mean = 63.95, SD = 17.91) compared to group with ADHD (mean = 56.98, SD = 16.04) but difference on the total test score was significant at one tail (t(168.776) = 1.918, $p < 0.057$). The computerised $\mu$ score for NC group was significantly correlated with the MABC-2 total score ($r = 0.26$, $p < 0.017$), balance score ($r = 0.34$, $p < 0.002$) and was determined by these factors (for the MABC-2 total score F(1,169) = 7.137, $MSE = 110.171$, $p < 0.008$ and for the balance score F(1,169) = 10.604, $MSE = 108.044$, $p < 0.005$) while for the ADHD group significant correlations were not found between computerised $\mu$ score and MABC-2 total score, or balance score. The paper and pencil $\mu$ score wasn't correlated with and determined by the MABC-2 scale scores for NC and ADHD groups.

### 4. Conclusions

In general: (1) the dual-task coordination is available in children/adolescents with ADHD in general and in its subtypes as in age and years of education matched NC; (2) Increase of the task difficulty in dual-task paradigm doesn't affect disproportionately children/adolescents with ADHD in comparison to age and years of education matched NC; (3) The paper and pencil version of the dual-task method is giving the same results in ADHD and NC as computerised version; (4) The dual-task functioning in ADHD in general and in its subtypes is not defined by the general motor functioning or by the comorbidity factors.

It can be concluded that dual task coordination is not distinguishing NC and ADHD and the subtypes of ADHD as was found in other studies [5,9]. It is proposed the separation of neural processes underlying the dual-task performance and executive dysfunction associated with ADHD. The study supports the concept of a separate cognitive function associated specifically with dual-task performance and provides evidence for the independence of the dual-task coordination function from the other executive functions that are compromised in ADHD.

### References

[1] A.D. Baddeley and S. Della Sala, Working memory and executive control, *Philosophical Transactions: Biological Sciences* **351**(1346) (1996), 1397–1404.

[2] R.A. Barkley, Behavioural inhibition, sustained attention, and executive functions: constructing a unifying theory of AD/HD, *Psychol Bull* **121** (1997), 65–94.

[3] G. Cocchini, R. Logie, S. Della Sala, S. MacPherson and A.D. Baddeley, Concurrent performance of two memory tasks: Evidence for domain-specific working memory systems, *Memory and Cognition* **30**(7) (2002), 1086–1095.

[4] K. Inasaridze, S. Della Sala and R.H. Logie, The Tbilisi Paper and Pencil Dual-Task, *Georgian Medical News* **150** (2007), 24–29.

[5] C. Karatekin, A test of the integrity of the components of Baddeley's model of working memory in attention-deficit/hyperactivity disorder (ADHD), *J Child Psychol Psychiatry* **45** (2004), 912–926.

[6] R. Logie, G. Cocchini, S. Della Sala, S. MacPherson and A.D. Baddeley, Is there a specific executive capacity for dual task coordination? Evidence from Alzheimer's disease, *Neuropsychology* **18**(3) (2004), 504–513.

[7] W. Pelham, E.M. Gnagy, K.E. Greenslade and R. Milich, Teacher ratings of DSM-III-R symptoms for the disruptive behavior disorders, *Journal of the American Academy of Child and Adolescent Psychiatry* **31** (1992), 210–218.

[8] J.A. Sergeant, H. Geurts and J. Oosterlaan, How specific is a deficit of executive functioning for Attention-Deficit/Hyperactivity Disorder? *Behavioural Brain Research* **130** (2002), 3–28.

[9] J. West, S. Houghton, G. Douglas and K. Whiting, Response inhibition memory, and attention in boys with attention-deficit/hyperactivity disorder, *Educational Psychology* **22** (2002), 533–551.
